# Beneficial effects of high-density lipoprotein (HDL) on stent biocompatibility and the potential value of HDL infusion therapy following percutaneous coronary intervention

**DOI:** 10.1097/MD.0000000000031724

**Published:** 2022-11-11

**Authors:** Jian-Di Liu, Ren Gong, Shi-Yuan Zhang, Zhi-Peng Zhou, Yan-Qing Wu

**Affiliations:** a Department of Cardiology, The Second Affiliated Hospital of Nanchang University, Nanchang, Jiangxi, China.

**Keywords:** high-density lipoprotein, percutaneous coronary intervention, stent biocompatibility

## Abstract

Several epidemiological studies have shown a clear inverse relationship between serum levels of high-density lipoprotein cholesterol (HDL-C) and the risk of atherosclerotic cardiovascular disease (ASCVD), even at low-density lipoprotein cholesterol levels below 70 mg/dL. There is much evidence from basic and clinical studies that higher HDL-C levels are beneficial, whereas lower HDL-C levels are detrimental. Thus, HDL is widely recognized as an essential anti-atherogenic factor that plays a protective role against the development of ASCVD. Percutaneous coronary intervention is an increasingly common treatment choice to improve myocardial perfusion in patients with ASCVD. Although drug-eluting stents have substantially overcome the limitations of conventional bare-metal stents, there are still problems with stent biocompatibility, including delayed re-endothelialization and neoatherosclerosis, which cause stent thrombosis and in-stent restenosis. According to numerous studies, HDL not only protects against the development of atherosclerosis, but also has many anti-inflammatory and vasoprotective properties. Therefore, the use of HDL as a therapeutic target has been met with great interest. Although oral medications have not shown promise, the developed HDL infusions have been tested in clinical trials and have demonstrated viability and reproducibility in increasing the cholesterol efflux capacity and decreasing plasma markers of inflammation. The aim of the present study was to review the effect of HDL on stent biocompatibility in ASCVD patients following implantation and discuss a novel therapeutic direction of HDL infusion therapy that may be a promising candidate as an adjunctive therapy to improve stent biocompatibility following percutaneous coronary intervention.

## 1. Introduction

High-density lipoprotein (HDL) is the smallest lipoprotein particle. Its main function in lipid metabolism is reverse cholesterol transport (RCT), wherein it attracts and collects cholesterol from peripheral tissues, such as arterial walls, and delivers it to the liver for eventual excretion.^[1]^ In fact, cholesterol carried by HDL has earned the moniker of “good cholesterol,” as considerable evidence suggests that HDL plays a protective role against the development of ASCVD.^[2,3]^ Epidemiological studies have indicated that the plasma concentrations of both HDL-C and the major HDL apolipoprotein, apoA-I, are independent, inverse predictors of the risk of having an ASCVD event;^[4–6]^ patients with pharmacologically controlled low-density lipoprotein levels and low HDL levels are still at an increased risk of ASCVD.^[7]^ Furthermore, Requena et al.^[8]^ have suggested that patients with a short-term, drug-induced decrease in HDL-C have a moderately increased long-term risk of cardiovascular events compared with those with constant HDL-C levels.

ASCVD, a prevalent disease worldwide,^[9]^ causes narrowing or occlusion of arteries (especially coronary arteries), thereby hampering myocardial perfusion. Therefore, percutaneous coronary intervention (PCI) with stent implantation to dilate the partly or fully occluded coronary artery lumen is an increasingly common treatment choice to improve myocardial perfusion in patients with ASCVD. Drug-eluting stents (DES) have been developed with the progress of stent design techniques. DES has decreased the incidence of in-stent restenosis (ISR) significantly, from 20% to 35% of bare metal stents (BMS) to about 10%, while also greatly reducing the revascularization ratio of target lesions. However, delayed endothelialization caused by locally delivered drugs from DES increases the risk of late and very late stent thrombosis (ST).^[10–12]^ Wenaweser et al.^[11]^ reported a 0.53% annual increase in the incidence of ST, a 3.3% cumulative incidence at 4 years, and a 5.7% rate of definite and probable ST after 4 years. Additionally, the ART-II trial showed a 9.4% rate of ST (definite, probable, or possible) among ASCVD patients with multiple vessel lesions at 5 years after DES implantation, while the 5-year major adverse cardiac and cerebrovascular event rate was 27.5%.^[13]^ Thus, the duration of dual antiplatelet therapy (DAPT) has been gradually extended from 1 month after bare metal stenting to 6–12 months or even longer after DES implantation.^[14,15]^ However, some patient populations with high bleeding risk are more prone to hemorrhagic complications with long-term DAPT (e.g., due to age, thrombocytopenia, concomitant use of oral anticoagulants, active cancer), so they would benefit from shortened DAPT duration to reduce the risk of bleeding complications.^[16]^ For those patients, the DAPT duration after PCI should be shortened to 1–3 months.^[17]^ Therefore, the optimal duration of DAPT after DES implantation is still under discussion. While clinicians worry that long-term DAPT would increase the risk of bleeding while preventing ST, they also worry that short-term DAPT may not be effective in the prevention of ST.

Multifactor regression analysis has identified stent re-endothelialization as one of the important factors that may reduce the incidence of ST. Hence, a question is raised as to how to promote stent re-endothelialization after DES implantation; thus, exploring the factors that improve stent biocompatibility following implantation is an important topic in the field of cardiology.^[18,19]^ This research will bring important clinical benefits to ASCVD patients, especially those at high risk of bleeding.

Previous reports have shown that re-endothelialization is associated with several factors, such as diabetes mellitus,^[20]^ baseline high-sensitivity C-reactive protein levels,^[21]^ plaque morphology,^[22]^ strut (design and material),^[23]^ drug elution (release kinetics),^[24]^ and coating polymer (material or degradation process).^[25]^ Although HDL is widely recognized as an essential antiatherogenic factor,^[26]^ there are few reviews about the effect of HDL on the improvement of stent biocompatibility after PCI.

This article focuses on the beneficial effect of HDL on stent biocompatibility in ASCVD patients following implantation and discusses a novel therapeutic direction for HDL infusion therapy.

## 2. Effects of HDL on endothelial cells (ECs)

The endothelium secretes many humoral factors that regulate vasodilatation and vasoconstriction of blood vessels, modulate platelet activation, coagulation, and fibrinolysis, and affect the proliferation and differentiation of smooth muscle cells (SMCs).^[27,28]^ Endothelial injury and dysfunction are the initial hallmarks in the pathogenesis of ISR and ST.^[29–31]^ One of the most important products of ECs synthesized in response to different physiological stimuli is nitric oxide (NO).^[28]^ Through the action of NO, the endothelium induces vasodilatation, attenuates thrombocyte adhesion and aggregation, and inhibits cell cycle progression of SMCs.^[28]^ Therefore, the integrity and function of the vascular endothelium are essential for the circulatory system. In this context, HDL has been reported as an important factor in sustaining endothelial function^[32–35]^ and protecting the endothelial structure.^[36,37]^

### 2.1. HDL protects endothelial function

**Figure 1.**
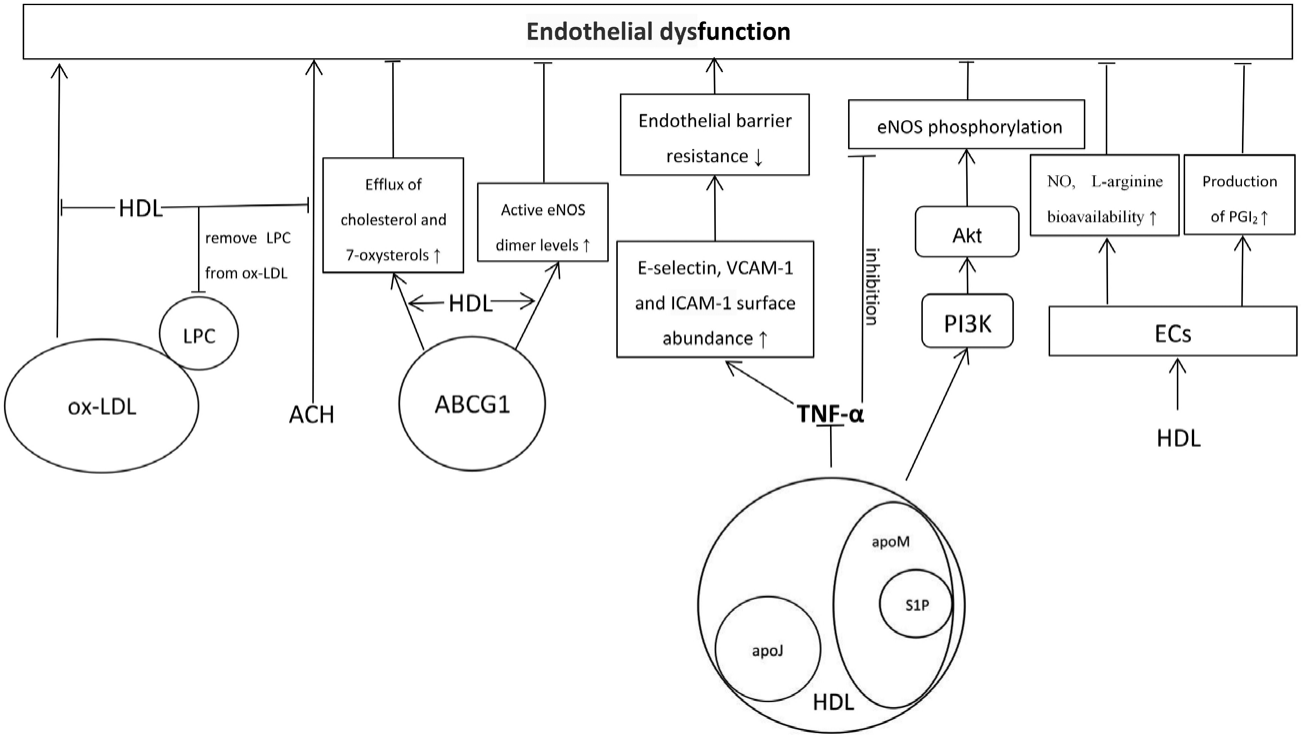
The effects of high-density lipoprotein on endothelial dysfunction. ABCG1 = ATP-binding cassette transporters G1, ApoJ = apolipoprotein J, ApoM = apolipoprotein M, ECs = endothelial cells, eNOS = endothelial nitric oxide synthase, HDL = high-density lipoprotein, ICAM-1 = intercellular cell adhesion molecule-1, LPC = lysophosphatidylcholine, NO = nitric oxide, ox-LDL = oxidized low-density lipoprotein, PGI_2_ = prostacyclin, PI3K = phosphatidylinositol 3-kinase, S1P = sphingosine-1-phosphate, TNF-α = tumor necrosis factor α, VCAM-1 = vascular adhesion molecule-1.

A previous study reported that HDL can reverse oxidized low-density lipoprotein (ox-LDL)-induced impairment of endothelium-dependent vasodilatation by preventing lysophosphatidylcholine from acting on the endothelium and removing lysophosphatidylcholine from ox-LDL.^[38]^ In vivo studies showed an inverse correlation between serum HDL concentration and abnormal vasodilatation induced by acetylcholine administered to the coronary arteries.^[39,40]^ In addition, decreased expression of endothelial nitric oxide synthase (eNOS) has been shown to be associated with endothelial dysfunction.^[41]^ Terasaka et al.^[42]^ suggested that HDL maintains endothelial function by promoting the efflux of cholesterol and 7-oxysterols and preserving active eNOS dimer levels via ATP-binding cassette transporter ATP-binding cassette transporter G1. Moreover, sphingosine-1-phosphate (S1P), which is carried by the apolipoprotein M-containing subfraction of HDL particles, can stimulate eNOS phosphorylation and NO production by activating the phosphatidylinositol-3-kinase/Akt/eNOS pathway in ECs.^[43]^ According to Kim et al,^[44]^ tumor necrosis factor α (TNF-α) considerably represses eNOS expression, but the inhibition can be restored by apolipoprotein J (apoJ), which is a protein component of HDL. Furthermore, Witting et al.^[45]^ found that serum amyloid A promoted endothelial dysfunction by decreasing NO and L-arginine bioavailability, but HDL pretreatment preserved overall endothelial function, suggesting that HDL may be protective. Moreover, previous studies have reported that isolated low HDL is associated with endothelial dysfunction, and rapid reconstituted HDL (rHDL) infusion results in a complete restoration of vasomotor responses to both serotonin and *N*^G^-monomethyl-L-arginine by increasing NO bioavailability.^[46–48]^ Also, HDL has a stimulatory effect on prostacyclin (PGI_2_) production by ECs.^[49,50]^ PGI_2_ has a vasorelaxing effect and diminishes the activation of platelets, and inhibits the release of growth factors, such as fibroblast growth factor, which stimulates proliferation of SMCs.^[51]^ However, a strong inflammatory component is involved in the pathogenesis of endothelial dysfunction. With the appearance of proinflammatory stimuli, ECs are activated and increase the abundance of adhesion molecules on their surfaces, such as E-selectin, vascular adhesion molecule-1, and intercellular cell adhesion molecule-1, which leads to the recruitment of proinflammatory immune cells to the vascular wall.^[52]^ Some in vitro studies^[44,53]^ have shown that HDL-associated S1P and apoJ significantly decrease the surface abundance of the 3 cell adhesion molecules through repression of the TNF-α/nuclear factor-κB (NF-κB) signaling pathway, suggesting that HDL/apolipoprotein M/S1P and apoJ not only maintain normal endothelial function under basal conditions^[54]^ but also maintain endothelial barrier integrity under inflammatory conditions. Therefore, the beneficial effect of HDL on endothelial function was remarkable (Fig. 1).

### 2.2. HDL promotes re-endothelialization

**Figure 2.**
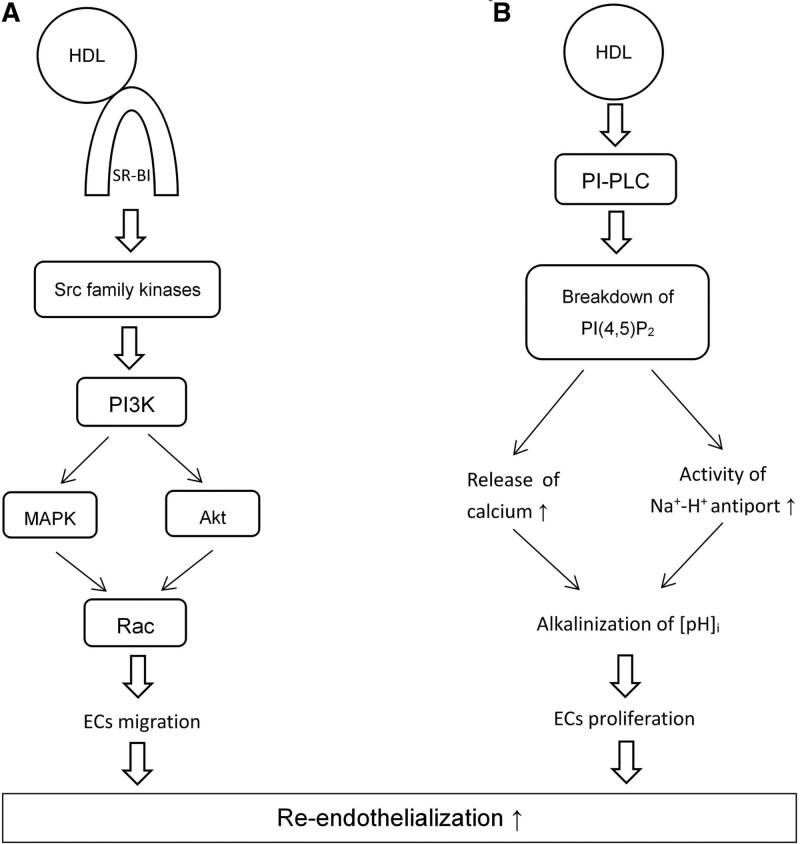
The mechanisms of high-density lipoprotein action on re-endothelialization. ECs = Endothelial cells, HDL = High-density lipoprotein, MAPK = Mitogen-activated protein kinase, [pH]_i_ = Intracellular pH, PI(4,5)P_2_ = Phosphatidylinositol-(4,5)-bisphosphate, PI3K = Phosphatidylinositol 3-kinase, PI-PLC = Phosphatidylinositol-specific phospholipase C, SR-BI = Scavenger receptor B type I.

Disintegration of the endothelium occurring after stent implantation induces the accumulation of platelets, the growth of SMCs, the chemoattraction of leukocytes, and several other processes, all of which ultimately lead to the occlusion of target vessels. Thus, the recovery of endothelial integrity is of immense importance in ASCVD patients following PCI treatment. It has been shown in vitro that HDL stimulates EC migration and promotes re-endothelialization in an NO-independent manner via scavenger receptor B type I-mediated activation of Rac GTPase (Fig. 2A).^[55]^ Tamagaki et al.^[37]^ investigated the effects of HDL on intracellular pH and on the proliferation of human vascular ECs. They showed that HDL promoted EC proliferation via the alkalinization of intracellular pH; the alkalinization effect was mediated by phosphatidylinositol-specific phospholipase C, which cleaves phosphatidylinositol-(4,5)-bisphosphate [PI(4,5)P_2_], thereby increasing the release of calcium from intracellular storage sites and activating the sodium–proton antiport (Fig. 2B).^[37]^ Additionally, Vanags et al^[56]^ found that apoA-I infusion increased the number of ECs in a murine model of stenting, suggesting that apoA-I promotes re-endothelialization via enhancement of endothelial progenitor cell mobilization.^[57]^

### 2.3. HDL reduces neointimal hyperplasia

**Figure 3.**
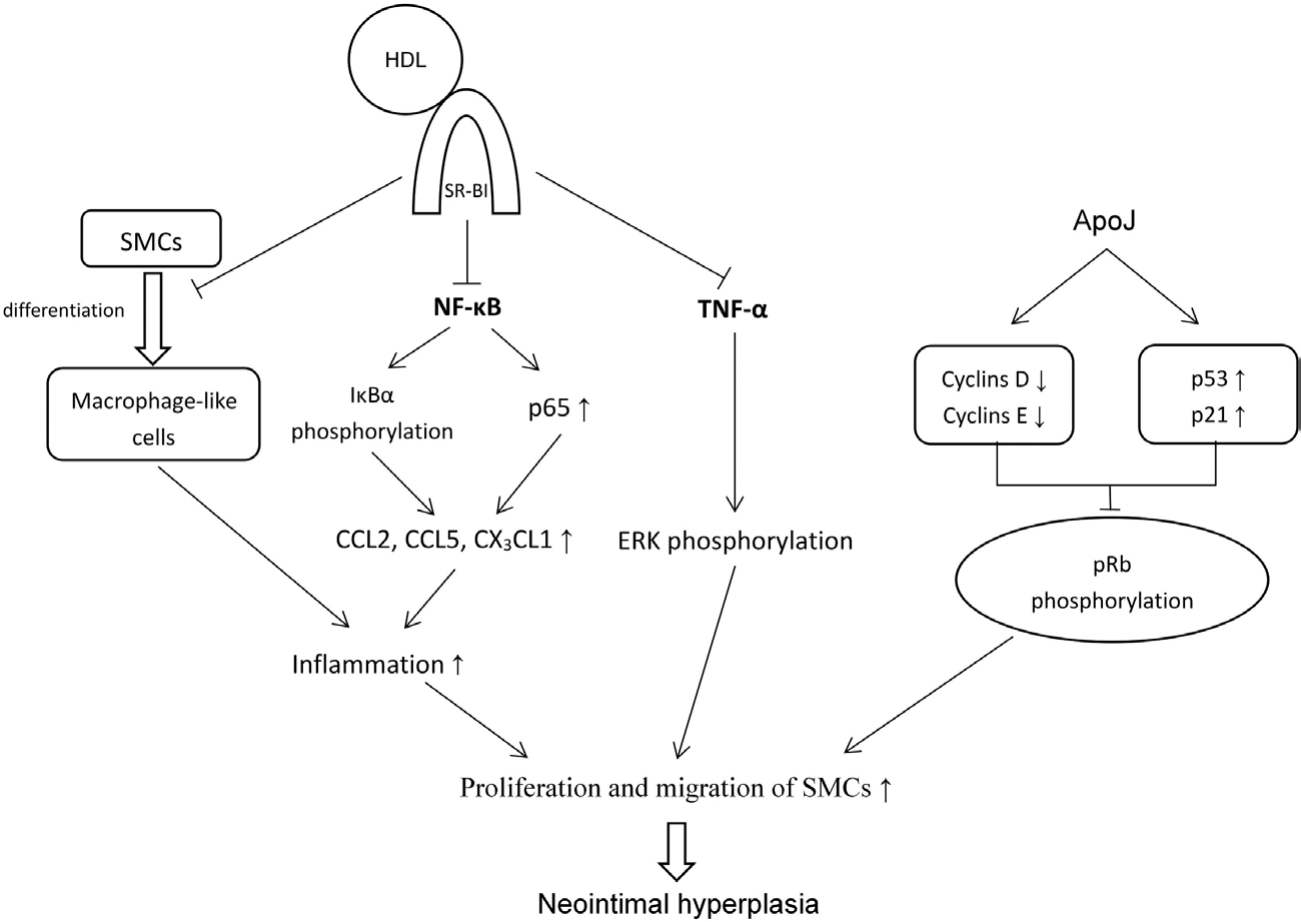
The effect of high-density lipoprotein on neointimal hyperplasia. ApoJ = apolipoprotein J, ERK = extracellular signal-regulated kinase, HDL = high-density lipoprotein, IκBα = inhibitor of NF-κB, NF-κB = nuclear factor-κB, PI3K = phosphatidylinositol 3-kinase, pRb = retinoblastoma protein, SMCs = smooth muscle cells, SR-BI = scavenger receptor B type I, TNF-α = tumor necrosis factor α.

Neointimal hyperplasia is characterized by the uncontrolled proliferation and migration of SMCs, which results in narrowing of the luminal area and eventual stent failure.^[58,59]^ Previous studies have shown that vascular SMC proliferation is enhanced by the inflammatory chemokines CCL2, CCL5, and CX_3_CL1.^[47,60–63]^ Some in vitro and in vivo studies have demonstrated that HDL can inhibit these chemokines^[64–68]^ by inhibiting the intracellular NF-κB pathways,^[69]^ and that it can also suppress SMCs proliferation directly by suppressing extracellular signal-regulated kinase phosphorylation.^[69]^ In addition, apoJ can inhibit SMC proliferation via induction of G1 cell cycle arrest accompanied by reduced retinoblastoma protein phosphorylation by downregulating cell cycle-promoting factors (cyclins D and E) and upregulating the p53–p21 (inhibitory proteins) pathway.^[44]^ Moreover, a previous study has reported that SMCs cause phenotypic changes regulated at the mRNA level in hyperlipidemic conditions, resulting in differentiation into macrophage-like cells; these effects are accompanied by a decreased expression of SMC α-actin.^[70]^ Given that macrophages promote the release of growth factors and cytokines that accelerate vascular SMC proliferation, they induce neointimal hyperplasia.^[71]^ Rong et al.^[70]^ suggested that cellular cholesterol content might play an essential role in determining the SMC phenotype, and α-actin^ + ^SMCs may diminish the inflammatory response in the formation of neointima. Another in vivo study demonstrated that apoA-I infusion reduces in-stent neointimal hyperplasia in a murine stent model.^[56]^ This finding suggests that apoA-I may preserve the SMC phenotype and prevent a switch into a more macrophage-like state by increasing cholesterol efflux from neointimal SMCs,^[70–72]^ which could create a less inflammatory environment and decrease the risk of ISR (Fig. 3).

### 2.4. HDL inhibits apoptosis of ECs

**Figure 4.**
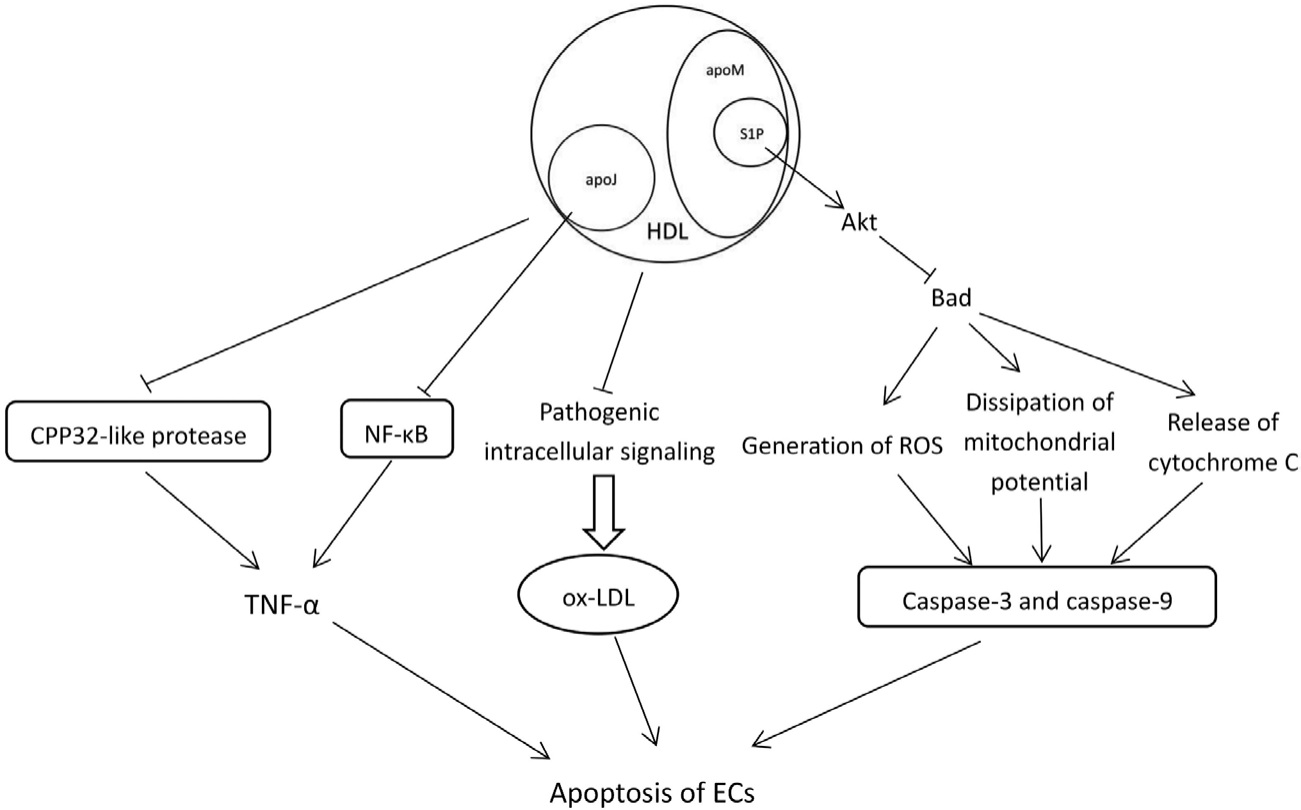
The effect of high-density lipoprotein on apoptosis of endothelial cells. ApoJ = Apolipoprotein J, ApoM = Apolipoprotein M, ECs = Endothelial cells, HDL = High-density lipoprotein, NF-κB = Nuclear factor-κB, ox-LDL = Oxidized low-density lipoprotein, ROS = Reactive oxygen species, S1P = Sphingosine-1-phosphate, TNF-α = Tumor necrosis factor α.

An in vitro study has shown that HDL prevents the apoptosis of ECs induced by TNF-α by inhibiting CPP32-like protease activity.^[73]^ Similar observations have been reported by Suc et al.^[36]^ and de Souza et al.^[74]^ who found that HDL increased the resistance of ECs against ox-LDL and prevented its toxic effect by blocking the pathogenic intracellular signaling (culminating in sustained calcium rise) involved in cell apoptosis. In addition, Zhang et al.^[75]^ suggested that HDL might significantly reduce the apoptosis of ECs via the suppression of caspase-3 activity. Furthermore, a study demonstrated that HDL protected ECs against growth factor deprivation-induced apoptosis, indicating that HDL and the associated S1P switched off the proapoptotic protein Bad via Akt stimulation, which led to the inhibition of the generation of reactive oxygen species, the dissipation of mitochondrial potential, and the release of cytochrome C into the cytoplasm, thereby preventing the activation of caspase-3 and -9 and apoptotic alterations of the plasma membrane.^[76]^ In addition, apoJ can diminish TNF-α-induced apoptosis of ECs by inhibiting the TNF-α/NF-κB signaling pathway.^[44]^ Both S1P and apoJ have been described as mediators of the anti-apoptotic activities of HDL towards ECs. (Fig. 4)

### 2.5. HDL protects ECs from the damage brought by the activation of complement system

ECs are exposed to activated complement during local or systemic inflammation, which causes membrane deposition of C5b-9 complexes.^[77]^ Perturbation of the plasma membrane by these proteins results in cell lysis or nonlytic alteration of cell function.^[78]^ Hamilton et al.^[79]^ reported that deposition of C5b-9 complexes on human ECs leads to an influx of extracellular calcium, activation of secretion of von Willebrand factor, and transient expression of P-selectin. Therefore, the activated complement system results in increased neutrophil, monocyte, and platelet adhesion, as well as increased thrombin generation, causing intravascular hypercoagulability, which increases the risk of ST.^[80,81]^ In addition, a clinical trial reported a significant increase in plasma levels of C5b-9 in patients with hypercholesterolemia compared with normoglycemic ASCVD patients and normal subjects, and the C5b-9 terminal complement complex levels were inversely correlated with HDL-C levels.^[82]^ In vitro studies have demonstrated that apoA-I and apoA-II inhibit complement complex-mediated cell lysis,^[83,84]^ because they can bind to the C9 complement factor and inhibit the formation of the C5b-9 terminal complement complex^[77,85]^ by interfering with the insertion of C9 into the lipid bilayer or with polymerization of C9 at C5b-8 sites.^[83]^ Additionally, apoJ is an inhibitor of the terminal complement complex, which inhibits C5b-9 terminal complement complex-mediated cell lysis in a concentration-dependent manner. It exerts an inhibitory effect by interacting with a structural motif common to C7, C8, and C9b.^[86]^ Thus, HDL may attenuate endothelial damage resulting from complement activation.

## 3. Effect of HDL on platelet activation

Given that platelet activation plays an important role in the formation of ST, prolonged DAPT results in a significant reduction in the rate of ST,^[87,88]^ and Naqvi et al.^[89]^ reported that HDL-C is a significant independent predictor of platelet-dependent thrombus formation. An epidemiological study has shown that low HDL levels are an important predictor of major cardiac events, including death, resulting from ST in patients following DES implantation.^[90]^ Therefore, researchers have suggested that HDL may inhibit platelet activation through various mechanisms.

A clinical trial has shown that platelet reactivity is significantly inhibited in rHDL-infused patients with diabetes mellitus via the reduction of P-selectin.^[91]^ This observation is in accordance with a study on a murine stent model that demonstrated that apoA-I infusion suppressed P-selectin activation.^[56]^ In vitro studies have shown that HDL suppresses adrenalin-, collagen-, ADP-, and thrombin-induced platelet aggregation,^[92–96]^ suggesting that this action is mediated by an increase in NO synthase activity in platelets.^[97]^ HDL also inhibits thrombin-induced fibrinogen binding and aggregation on platelets by inhibiting phosphatidylinositol 4,5-bisphosphate turnover, 1,2-diacylglycerol and inositol 1,4,5-trisphosphate formation, and intracellular calcium mobilization.^[98]^ Desai et al.^[99]^ demonstrated that HDL impairs platelet responsiveness to exogenous agonists via occupation of cell-surface receptors by HDL-E particles. Moreover, Sugatani et al.^[100]^ found that HDL reduces the accumulation of platelet-activating factor by inhibiting platelet-activating factor synthesis, which is mediated via the suppression of acetyl-CoA:1-alkyl-2-lyso-sn-glycero-3-phosphocholine acetyltransferase activation. In addition, HDL prevents platelet hyperreactivity by limiting intraplatelet cholesterol overload, but also by modulating platelet signaling pathways after binding to the platelet HDL receptors scavenger receptor B type I^[101]^ and apoE receptor 2.^[99]^ Moreover, HDL stimulates the endothelial production of NO^[102]^ and PGI_2_,^[49,50]^ which are potent inhibitors of platelet activation.^[28,51,103]^

## 4. Discussion

HDL can sustain vascular endothelial function, enhance re-endothelialization, inhibit neointimal hyperplasia, protect endothelial integrity, and reduce inflammatory response and platelet activation, indicating that it plays an important role in the prevention of ISR and ST. Thus, HDL is important for ASCVD patients, especially for those who undergo PCI treatment with stent implantation.

Unfortunately, decreased serum levels of HDL-C are commonly encountered in ASCVD patients,^[104]^ and experts have reached a consensus on the point that low serum HDL-C (<1.0 mmol/L) is an independent risk factor for ASCVD.^[105,106]^ Thus, the use of HDL as a therapeutic target has been of great interest. After 3 orally active HDL-raising agents, including niacin^[107,108]^ and 2 cholesteryl ester transfer protein inhibitors,^[109,110]^ failed in prospective intervention trials, experts proposed a change in the target of HDL therapy from elevation of circulating HDL-C levels to promote the functional properties of HDL.^[111,112]^ Thus, the focus shifted to HDL infusion therapy, which can transiently increase the number of HDL particles and thereby enhance RCT.^[111,112]^ HDL infusion agents include partially delipidated, isolated HDL proteins, and native apoA-I or genetic variants.^[113]^ These agents are classified as either reconstituted or recombinant, where rHDL is derived from human plasma, while recombinant is formed using other sources.^[114]^ According to previous studies, 3 important HDL formulations have been clinically evaluated. The first agent is MDCO-216 (and its precursor ETC-216), also known as apoA-I_Milano_, which is a naturally occurring genetic mutation in apoA-I. This variant has been found to have a shortened lifetime in the plasma, which causes faster catabolism of apoA-I, thereby increasing the amount of lipid-poor apolipoprotein present in plasma and increasing RCT capabilities.^[115]^ Thus, recombinant apoA-I_Milano_ was developed for infusion. Small intravascular ultrasound (IVUS) clinical trials (47–60 patients) have compared the effect of ETC-216 or placebo on coronary atheroma burden, and showed that infusions of apoA-I_Milano_ in patients with coronary artery disease significantly reduced coronary plaque volume (1%–2% relative to placebo) and were safe and generally well tolerated.^[116,117]^ However, a double-blind, randomized, multicenter trial has compared the effects of 5 weekly intravenous infusions of MDCO-216 at a dose of 20 mg/kg weekly (n = 59) with placebo (n = 67) in statin-treated patients with acute coronary syndrome (ACS). The results showed that MDCO-216 infusion did not produce an incremental plaque regression.^[118]^ The second agent is CER-001, which is an artificial HDL-mimetic composed of human recombinant human apoA-I and 2 naturally occurring phospholipids. In a smaller human study, patients with familial hypoalphalipoproteinemia were given 20 infusions of CER-001.^[119]^ After only 9 infusions, magnetic resonance imaging showed a significant increase in mobilization of cholesterol from the arterial wall. Six months after infusions, there were significant increases in the amounts of apoA-I, HDL, and free cholesterol.^[119]^ A clinical trial compared the effect of 6 weekly infusions of CER-001 (3, 6, and 12 mg/kg) versus placebo on coronary atherosclerosis in 369 ACS patients using IVUS, and found that infusions of 3 mg/kg CER-001 induced the greatest atheroma regression in ACS patients with higher baseline percent atheroma volume.^[120]^ Nevertheless, the results of a prospective, double-blinded, randomized trial conducted at 51 centers comparing the effect of 6 weekly infusions of CER-001 (3, 6, and 12 mg/kg) versus placebo in 570 ACS patients showed that CER-001 infusions did not reduce coronary atherosclerosis.^[121]^ Another double-blind, randomized, multicenter trial compared the effect of 10 weekly infusions of CER-001 (3 mg/kg) (n = 135) versus placebo (n = 137) in ACS patients with a high plaque burden, and the results demonstrated that infusion of CER-001 did not promote regression of coronary atherosclerosis.^[122]^ The third agent is CSL112 (and its precursor CSL111), which contains reconstituted formulations of human plasma-derived apoA-I and phosphatidylcholine to form synthetic HDL particles. Clinical trials have assessed the safety and pharmacokinetics/pharmacodynamics of CSL112 infusion in patients with stable atherosclerotic disease.^[123,124]^ The results showed that CSL112 infusion was not only well tolerated but also immediately raised apoA-I levels and caused a rapid and marked increase in the capacity of serum to efflux cholesterol.^[123,124]^ In a phase II study to further evaluate the efficacy, patients were randomized to receive either CSL112 or placebo, and efficacy was assessed using IVUS and coronary angiography.^[125]^ Although there was no statistically significant difference between atheroma volume in CSL112 versus placebo after 4 weekly infusions, both the plaque characterization indexes and the coronary score on angiography (indexes to measure the composition of the plaque and quantify the burden of coronary artery disease, respectively) showed significant decreases compared with placebo.^[125]^ HDL infusion therapy can induce an acute increase in the plasma concentrations of apoA-I,^[126,127]^ although the effect duration is relatively short, given that the half-life of apoA-I is approximately 48 to 72 hours.^[114]^ It has been suggested that the intravenous administration of HDL infusion therapy is unsuitable for long-term treatment regimens because liver toxicity (as indicated by elevation of transaminases) has been observed at a higher rHDL infusion concentration in early phase trials of CSL-111.^[128]^ However, further studies have reported that the reformulation of HDL infusion agents (CSL-112) is well tolerated and safe, without evidence of any major organ toxicity,^[123,125]^ indicating that the toxic effect of CSL-111 can be attributed to the excipients rather than the apoA-I component. In summary, the development of MDCO-216 and CER-001 has been discontinued because of a lack of efficacy in plaque regression in clinical trials.^[129]^ However, CSL112 stimulates a far more substantial increase in ABCA1-dependent cholesterol efflux capacity than that achieved in phase II studies of MDCO-216 and CER-001 (330% vs. 80%–90% and 6%, respectively), which shows a heady prospect.^[124]^ We believe that some negative studies do not indicate the end of the research on apoA-I-based therapeutics. We look forward to the results of the AEGIS-II phase III study.^[130]^ Perhaps this large clinical trial will confirm the unique therapeutic value of CSL112.

However, previous animal studies and clinical trials related to HDL infusion therapies have focused on the stabilization of plaques and regression of atherosclerosis,^[119,121,131–134]^ while few studies have focused on the value of HDL infusion therapy as an adjunctive therapy to promote post-PCI recovery of target vessels. To date, only Vanags et al^[56]^ and Kaul et al^[135]^ have reported the potential value of HDL infusion following BMS implantation. Although the 2 studies were animal studies and not clinical trials, the results were groundbreaking.

Over the last 2 decades, improvements in interventional techniques, refinements in stent design (particularly the advent of DES), and adjunctive DAPT have resulted in a remarkable reduction in the overall rates of stent failure. However, although the technology of DES design continues to improve, unresolved problems related to stent biocompatibility persist, including delayed re-endothelialization and neoatherosclerosis, which cause ST and ISR.^[136]^ Although first-generation DES were efficacious in reducing ISR compared with BMS, they resulted in an increase in ST.^[137]^ Vascular toxicity from the polymers that were not adequately biocompatible, delayed re-endothelialization, and ongoing inflammation were the most common causes of this phenomenon.^[137]^ Second-generation DES with more biocompatible polymers, thinner, more flexible cobalt-chromium or platinum-chromium struts, and newer anti-proliferative drugs – with the 2 limus analogs (zotarolimus and everolimus) replacing paclitaxel to exhibit a wider toxic-therapeutic ratio—have markedly reduced but not eliminated ST.^[138]^ Furthermore, neoatherosclerosis is an important contributing factor to late stent-related cardiovascular events after DES deployment. The histopathological substrate of neoatherosclerosis is similar to that of native atherosclerosis, which contains macrophage/foam cells, cholesterol clefts, areas of calcification, and necrotic cores.^[139]^ It has been widely hypothesized that the underlying biological mechanisms leading to neoatherosclerosis are closely related to the progression of native coronary atherosclerosis.^[140]^ During stent deployment, as the vascular wall undergoes expansion by stent struts, endothelial denudation, significant medial injury, plaque compression, and rupture of the internal elastic lamina occur, thereby triggering an inflammatory response. Over time, plaque is capable of becoming a source of growth factors, cytokines, and chemokines, thereby promoting neoatherosclerosis.^[140]^ Neoatherosclerosis occurs within a much shorter time frame than native atherosclerosis at 6 months to 5 years after stent deployment.^[141]^ Neoatherosclerosis accelerates the late expansion of the neointima as a key cause of stent failure. Neointimal plaques can also become unstable, with ruptured thin-capped neointimal plaques acting as the primary cause of very late ST.^[142]^ Although the mechanisms of neoatherosclerosis have not been entirely elucidated, the higher occurrence of neoatherosclerosis in DES may be the result of drug resistance, a reaction to the DES polymers, or DES-induced delayed re-endothelialization.^[142]^ Therefore, the addition of novel adjunctive therapies to reduce these risks remains crucial.

Numerous previous studies have documented the beneficial effects of HDL on blood vessels^[56,91,127]^ and the safety of HDL infusion therapies,^[123,125]^ suggesting that the HDL infusion may be a promising therapy to improve stent biocompatibility for ASCVD patients with DAPT intolerance after stenting. However, no clinical trials thus far have demonstrated the benefits of HDL infusion for ASCVD patients who underwent PCI treatment. Large clinical trials of HDL infusion should be conducted in these patients; however before that, researchers have to make a very detailed proposal about the single dose and treatment duration of HDL infusion therapy after DES implantation. Overall, HDL formulations delivered via infusion represent a new modality of adjunctive therapy following PCI, which is a novel research direction.

## 5. Conclusion

HDL has a number of beneficial effects on stent biocompatibility after PCI, such as the maintenance of vascular endothelial function, protection of endothelial integrity, enhancement of re-endothelialization, and reduction of inflammation and platelet activation. HDL infusion therapy is a promising candidate for improving stent biocompatibility following implantation. We believe that the application of HDL infusion therapy following DES implantation will greatly shorten the duration of DAPT and significantly reduce the incidence of ST. Given that there are few related studies, further studies on HDL infusions and the beneficial role of HDL in stent biocompatibility have the potential to yield better adjunctive therapy regimens following PCI.

## Author contributions

Conceptualization: Jian-Di Liu, Yan-Qing Wu.

Data curation: Jian-Di Liu, Ren Gong, Shi-Yuan Zhang, Zhi-Peng Zhou.

Writing – original draft: Jian-Di Liu.

Writing – review & editing: Jian-Di Liu, Yan-Qing Wu.
